# Inspirations of traditional Chinese martial arts Qinggong training for parkour

**DOI:** 10.3389/fspor.2026.1855244

**Published:** 2026-08-12

**Authors:** Jianbin Zhang, Yating Zhan, Lilan Wu, Tianyu Liu, Zhi Li

**Affiliations:** 1 School of Physical Education and Health, Chengdu University of Traditional Chinese Medicine, Chengdu, Sichuan, China; 2 School of Chinese Classics, Chengdu University of Traditional Chinese Medicine, Chengdu, Sichuan, China; 3 School of Physical Education, Southwest University of Finance and Economics, Chengdu, Sichuan, China

**Keywords:** movement pedagogy, obstacle negotiation, parkour, qinggong, skill progression

## Abstract

Qinggong and parkour emerged in different cultural-historical settings, yet both address the practical problem of negotiating obstacles efficiently. This study examines how historically situated Qinggong training ideas might serve as heuristic resources for contemporary parkour pedagogy. A cross-cultural conceptual comparative design was applied to a purposively and theoretically sampled corpus comprising Qinggong and Wushu manuals and historical texts, contemporary Chinese Wushu scholarship, parkour sociocultural and pedagogical literature, biomechanical, physiological, and injury-related studies, and relevant movement-science research. Sources were compared in terms of movement problems, technical categories, landing and impact management, balance and environmental adaptation, physical and perceptual demands, and principles of skill progression. The comparison identifies functional and pedagogical correspondences in movement continuity, landing control, constrained-support balance, variable-environment practice, repetitive training, and gradual increases in task difficulty. Five testable applications are consequently proposed for formal parkour coaching: using landing sound as feedback; diversifying fall-management drills; manipulating support-surface and visual constraints; introducing carefully monitored loading–unloading sequences; and sequencing prerequisite, specialized, and integrative tasks. These applications are synthesized in a foundation–specialization–practice framework that progresses from movement literacy and risk management to modular skill development and representative obstacle routes. The identified correspondences provide a theory-building and hypothesis-generating model whose transferability requires biomechanical testing and pedagogical intervention research across age groups, training levels, and cultural settings.

## 1 Introduction

Since the 21st century, an emerging urban movement practice emphasizing free movement through obstacles, known as parkour, has risen globally. Parkour adepts, known as “traceurs” (from the French verb “tracer”, which means both going fast, and drawing a line) (1), utilize their physical capabilities to traverse urban environments quickly. When discussing obstacle-crossing movements in China, traditional Chinese martial arts Qinggong is frequently referenced. Qinggong refers to the development of swift and agile movement capabilities through specialized training, enabling enhanced obstacle-crossing performance (2). Qinggong literally combines “Qing”, denoting lightness or agility, and “Gong”, denoting cultivated skill or trained capacity; it should therefore be understood as disciplined skill in light, agile movement rather than as supernatural ability (2, 3). Some later Qinggong descriptions are preserved in Shaolin-related manuals; these are treated as one part of a diverse textual corpus (4, 5).

Looking across parkour and Qinggong, both aim for the rapid and efficient overcoming of obstacles, emphasizing the coordinated use of agility, strength, and body control. However, their training systems emerged from different cultural-historical contexts. Current academic research on Qinggong and parkour remains largely field-specific. Comparative studies linking the two are limited, and associated theoretical and practical explorations are relatively scarce.

Movement techniques in both are not merely biological actions but socially transmitted ways of using the body, through which societies organize posture, rhythm, effort, perception, and skill acquisition (6). Qinggong and parkour are therefore treated here as culturally situated systems of embodied knowledge. Spatz's account of technique as embodied knowledge further helps to frame the comparison as a study of reconfiguration and pedagogical possibility (7). Qinggong is therefore not treated as a single unified tradition, but as a historically variable cluster of training ideas, techniques, ideals, and representations shaped by martial, performance, literary, and pedagogical contexts. On this basis, parkour is treated as a contemporary movement culture rather than solely a competitive sport, while Qinggong is read as a historically layered body culture preserved in manuals, martial pedagogy, and later cultural representations; the analytical value of the comparison lies in heuristic translation across movement cultures (8).

The core objective of this paper is to conduct a conceptual comparison and systematic deconstruction of the training systems and technical characteristics of Qinggong and parkour. The comparison is organized around functional analogy and pedagogical heuristic. Qinggong sources are used to identify historically situated movement ideals, training progressions, and embodied principles, while parkour studies are used to clarify contemporary movement categories, coaching concerns, and available biomechanical evidence. Accordingly, this paper asks three connected questions: (1) how comparable obstacle-crossing problems are addressed in Qinggong and parkour; (2) what technical and training correspondences can be identified; and (3) how these correspondences may generate testable pedagogical hypotheses for an integrative parkour training pathway.

## 2 Research design and methods

### 2.1 Research design

This study is designed as a cross-cultural conceptual comparative analysis. Its purpose is to examine how historically situated descriptions of Qinggong and contemporary parkour scholarship can be brought into a controlled comparison that generates analytically defensible pedagogical hypotheses. The comparison therefore proceeds from functional analogy and interpretive correspondence.

### 2.2 Source corpus and sampling strategy

The source corpus comprised five categories: traditional Qinggong and Wushu manuals or historical texts; contemporary Chinese Wushu scholarship; parkour sociocultural and pedagogical literature; parkour biomechanical, physiological and injury-related studies; and broader movement-science literature on landing, balance, respiratory-trunk stabilization and staged skill development. The Qinggong corpus was used to identify technical ideals, training progressions and culturally situated pedagogical norms, while the parkour corpus was used to identify contemporary movement categories, coaching concerns and empirically examined mechanisms.

Materials were selected through purposive and theoretical sampling. Sources were included when they directly addressed one or more of the following analytical concerns: Qinggong or Wushu training, obstacle-crossing movement, jumping and landing, climbing or vaulting, parkour movement classification, parkour pedagogy, landing biomechanics, balance control, respiratory or trunk stabilization, and staged skill development. The aim was conceptual relevance to the research questions, not exhaustive coverage of all texts associated with Chinese martial arts or all studies on parkour.

### 2.3 Purposive Sampling and Eligibility Criteria

The analysis distinguished between four evidentiary functions: empirical evidence, historical-textual evidence, conceptual translation and interpretive hypothesis. Empirical claims were drawn only from studies reporting measured data or clearly identified research findings. Qinggong manuals, martial arts narratives, historical references and later reconstructions were treated as historical documents that can reveal technical ideals, pedagogical norms and symbolic values, but not as direct proof of technical efficacy, historical transmission or modern pedagogical transfer. Fictional, cinematic, mythologized or unsupported practitioner claims were excluded from technical evidence, although they may be mentioned as cultural representations where relevant.

The comparative procedure proceeded in three steps. First, movement problems were identified in both corpora, including obstacle crossing, landing, balance, climbing, vaulting and staged progression. Second, relevant passages were extracted based on the movement issues, evidence, technical categories, training principles and potential pedagogical applications described therein, and were then compared individually. Third, potential parkour applications were reformulated as pedagogical hypotheses with observable variables and boundary conditions.

### 2.4 Translation and terminological treatment

Chinese-language materials were read in the original, and English renderings of key terms were finalized manually by the authors. No automatic translation tool was used to determine key analytical terms. When no direct English equivalent could adequately carry the cultural and technical meaning of a Chinese term, the translation combines transliteration or literal rendering with functional explanation.

## 3 Overview of Qinggong in traditional Chinese martial arts

### 3.1 Origin and historical evolution of Qinggong

In early human hunting and migration, people gradually mastered skills such as efficient climbing, endurance running, and jumping to obtain food and avoid danger (9, 10). These techniques, derived from survival practices, were later consciously applied in military contexts (11). In ancient warfare, in order to scale city walls for reconnaissance and attack, soldiers were required to possess agility that exceeded that of ordinary people; such practical combat demands gave rise to the early prototype of Qinggong (2). From the Pre-Qin period through the Qin and Han dynasties, specific political demands led to the application of such techniques among assassins, and numerous records emerged about individuals adept at climbing and leaping over roofs (12, 13). From this perspective, the emergence of Qinggong bears a profound imprint of survival.

With changes in the social environment, this type of skill gradually moved into civil society, where it was utilized for tasks such as delivering messages, pursuing targets, or escaping danger, and ultimately evolved into an important component of traditional martial arts. The Ming and Qing dynasties constituted a flourishing stage of martial arts. During this period, Qinggong techniques were further refined and categorized, forming diverse methods of practice and schools. Successive martial arts classics (4, 14, 15) document these developments in detail. These historical records delineate the technical categories, training ideals, and pedagogical norms of Qinggong, including “Flying Skill”, “Pit-Jumping Skill”, “Board-Running Skill”, “Stake-Stepping Skill”, “Brick-Stepping Skill”, “Method of Leaping over Roofs and Walking on Walls”, “Vertical Leaping Technique”, and “Water-Walking”, among others.

During the Qing dynasty, with the extensive use of firearms and artillery on the battlefield, the military function of traditional martial arts was greatly reduced (16). Qinggong, which had once flourished, also gradually declined and began to be preserved in the form of performance and cultural symbolism. As early as the Ming and Qing periods in folk society, Qinggong techniques had already been incorporated into performing arts such as opera and acrobatics. The flips, leaps, pounces, and falls performed by martial-role actors on the opera stage, as well as stilts and lion dance in folk performances, all reflect the skillful application of Qinggong techniques (17, 18). At the same time, the rise of martial arts fictions in the late Qing dynasty and early Republic of China also exerted a significant cultural reshaping effect on the image of Qinggong. In these works, heroes depicted frequently leaping across roofs and running on walls, traversing through the air, and even reaching the exaggerated realm of “leaving no trace upon the snow” (19). However, the public has come to understand Qinggong largely through fictions and opera, which has contributed to some mythologized misunderstandings of it. During the Republican period, in order to cater to the social yearning for extraordinary martial arts skills, martial arts sects often integrated the Qinggong plots from fictions with martial arts performances; consequently, Qinggong was formally packaged and debuted as an integral part of martial arts (2, 20).

Subsequently, under the influence of scientific thought in the mid-20th century and with the development of empirical research, people began to examine Qinggong rationally and to reconsider the legends imbued with supernatural elements. Researchers, through the verification of historical documents and oral traditions (2, 21), clarified that Qinggong is not an elusive magical art, but rather the result of traceable physical training.

### 3.2 Concept and scope of Qinggong

In Wushu (3), Qinggong is also referred to as jumping skill, which denotes the enhancement of lower-limb jumping ability through various specialized training methods, enabling practitioners to jump higher and farther. Practicing Qinggong does not change a person's body mass; rather, it enables practitioners to perform obstacle-crossing movements with a lightness and agility that untrained individuals typically cannot achieve.

From a categorical perspective, in modern broad terms, Qinggong can be divided into the following three related domains (2). The first is Qinggong in traditional martial arts, that is, the kung fu passed down by generations of martial artists. The second is obstacle-crossing and climbing training in military and police training systems, which shares principles similar to those of Qinggong (22). The third is various traversal movements in parkour.

### 3.3 Core technical composition and training methods of Qinggong

The Qinggong technical system mainly comprises three elements—force, balance, and buffering (22)—and includes eight movement categories: running, jumping, darting, bouncing, vaulting, leaping, climbing, and crawling (3).

Qinggong training places particular emphasis on the coordinated control of “speed and stability” as well as “takeoff and landing” (3). Qinggong masters pay great attention to the practice of “silent landing” (5); this control ability not only reflects a practitioner's high degree of mastery over their own body, but is also a necessary condition for avoiding sports injuries and achieving smooth and continuous movements.

In Chinese traditional martial arts, there are various training methods for Qinggong, and generations of martial artists often simulate real movement scenarios by skillfully designing training environments. One such training method in traditional martial arts is the “Plum-Flower Stakes” practice, in which practitioners jump and stand on stakes of different heights and distances to train their balance and reactive abilities.

Qinggong training follows a progressive principle—“from stillness to movement, from easy to difficult, and from low to high”—emphasizing gradual, staged skill development. The beginner stage focuses on the cultivation of static strength, such as basic exercises like standing in the horse stance and coordinating the waist and legs, in order to enhance lower-limb strength and body stability, laying a solid foundation for the “movement” stage. With the consolidation of foundational skills, training enters the dynamic stage, with speed and complexity continuously increasing so that the body gradually adapts to the demands of high-speed running and leaping. The principle of “from low to high” is reflected in the gradual progression of challenges in height and distance. Throughout the process, each advancement is built upon the body's adaptation to the previous stage. Ultimately, this training ideal seeks to integrate speed, strength and skill, enabling practitioners to move lightly and respond flexibly when facing various obstacles.

## 4 Analysis of functional correspondences between Qinggong and Parkour

### 4.1 Functional objectives and movement demands

Both Qinggong and parkour share the core objective of navigating obstacles in complex environments in a practical and efficient manner. This pursuit determines that the two have similar requirements for human physical functions.

In terms of specific functional pursuits, both practices embody a goal orientation toward movement efficiency. Existing literature points out that parkour overlaps with related practices such as the Art du Déplacement and free running; however, while the latter emphasize aesthetics and personal expression, the core philosophy of parkour is often described as pragmatic movement through obstacles, which is one key distinction between parkour and activities that focus more strongly on performance and expression (1, 8, 23, 24). Correspondingly, Qinggong have consistently revealed a practical orientation throughout their historical origins and development, being closely linked to actual needs in survival, military and civilian contexts (2, 11–13). In this sense, the “Qing” pursued by Qinggong is regarded as a historically formed training ideal, aimed at enabling practitioners to move swiftly, steadily and with minimal unnecessary exertion in challenging spatial environments.

In terms of movement demands, empirical parkour studies show that practitioners use strategies such as forefoot landings, rolls, and increased lower-limb joint flexion to prolong deceleration time, distribute impact across the kinetic chain, and maintain movement continuation after landing (25–27). Similarly, Qinggong training descriptions emphasize accomplishing greater spatial displacement with limited physical energy reserves. Through long-term systematic training, Qinggong sources describe the coordination between muscular explosive power, and stride rhythm as a basis for agile movement (28). In practice, movements such as wall ascent, ditch crossing and hurdle clearance are described as being completed with few support points and short pause times, so as to support sustained action under high-intensity task conditions (22).

### 4.2 Technical correspondences in movement structure

Parkour's movement system is organized into six major categories: tumbling, hanging, balance, climbing, jumping, and hybrid (combination) movements (29). Narrative and biomechanical studies further show that parkour commonly involves running, jumping, vaulting, climbing, rolling, balancing, and adapting bodily movement to non-standardized environmental obstacles (1, 25–27).

Wushu and Qinggong-related texts describe Qinggong as a training system concerned with running, jumping, darting, bouncing, vaulting, leaping, climbing, crawling, balance control, take-off and landing coordination, and progressive obstacle-crossing practice (2, 3, 22). Historical and traditional martial arts materials further record concrete skill names and training forms such as “Pit-Jumping Skill”, “Stake-Stepping Skill”, “Brick-Stepping Skill”, “Vertical Leaping Technique”, wall-stepping and wall-leaping methods, rolling and falling techniques, and progressive loading or obstacle-based exercises (4, 5, 30, 31). Table 1 was compiled from the Qinggong/Wushu and parkour sources cited in this section.

**Table 1:** Analytical comparison of technical movements between Qinggong and parkour.

**Analytical grouping**	**Qinggong Techniques**	**Parkour Terminology**	**Functional Objective**	**Relevant Biomechanical Mechanisms**
**Jumping & Leaping**	Stride leap; Precision leap; Rotational leap; Step-drop landing; Caliper jump; Suspended rebound leap; Bar release jump.	Precision jump; Running precision; Stride (bounding); Drop landing; Cat leap.	Cross horizontal gaps and vertical height differences while ensuring landing precision and movement continuity.	Hip–knee–ankle triple extension, center-of-mass trajectory control and eccentric buffering to absorb ground reaction forces.
**Support-Assisted Rebound**	Wall-, platform- and pole-assisted rebound; Hybrid rebound.	Tic-tac; Wall run; Pole push; Step vault variants.	Use brief support contacts to redirect horizontal momentum into upward or lateral displacement.	Impulse generation, friction/reaction-force coordination and support-angle- control.
**Vaulting & Grasping**	Bar, eave-edge, cylindrical and one-arm grasps; suspended release/catch movements.	Kong/monkey vault; Cat grab; Lache; Underbar; One-arm catch.	Create an upper-limb anchoring point during aerial traversal or suspension transition.	Shoulder girdle stabilization, grip force production, angular momentum regulation, impact absorption and redistribution.
**Climbing & Overcoming Obstacles**	Building climbing; Single-story ascent; Low/mid/high wall crossing techniques.	Climb up; Wall climb; Cat-to-cat; Fence vault; Climb-over techniques.	Traverse vertical architectural barriers and shift between spatial levels.	Lower-limb propulsion, upper-limb pulling and sequential grab-pull-step-vault coordination.
**Balance**	Leg-only, arm-assisted and quadrupedal traversal on constrained supports.	Rail balance; Precision balance; Cat balance; Beam walking.	Maintain dynamic and static stability on narrow or elevated support surfaces.	Vestibular–proprioceptive integration; Core adjustment; Fine foot-arch control.
**Impact Dissipation & Landing**	Layered drop descent; Eave-assisted height reduction; Rolling, side-fall and backward-fall methods.	Parkour roll; Dive roll; Shoulder roll; Controlled landing; Breakfall.	Reduce landing impact and preserve transition into subsequent movement.	Impulse prolongation; Eccentric absorption; Vertical-to-rotational momentum conversion.
**Composite Skill**	Hybrid rebound; Integrated climbing sequences.	Flow sequences; Linked movement patterns; Obstacle sequences.	Achieve seamless multi-movement integration within complex environments.	Kinetic chain energy continuity; Spatiotemporal rhythm; Task-specific neuromuscular coordination.

Note. The Qinggong column reports textual training categories and skill descriptions; the parkour column reports co*n*temporary movement terminology. The biomechanical mechanisms are interpretive summaries used to clarify comparable functional movement demands and should not be read as direct empirical evidence for Qinggong efficacy.

### 4.3 Interpretive parallels in movement and biomechanical mechanisms

From the perspective of movement technique, Qinggong and parkour can be read as addressing comparable movement-chain problems. The complete landing action process in parkour constitutes a continuous movement chain composed of “approach run, take-off, aerial phase, and landing” (25). Similarly, the Qinggong notion of “lifting ‘Qi’ to lighten the body” (2, 22), can be understood as building up power by controlling one's breathing during the run-up, and then explosively releasing that power upon take-off. From movement-science perspective, this cue can be interpreted as directing attention to breath coordination, anticipatory trunk organization, and timing of power release (32, 33).

The landing phase offers one of the clearest functional parallels. Qinggong texts emphasize not only jumping high but also landing stably (22). In parkour, experienced practitioners use joint flexion and eccentric contraction to absorb impact (26). In both cases, landing is described or measured as a lower-limb control problem in which deceleration time, impact absorption and post-landing continuity are central concerns.

In summary, the two practices can be read as addressing comparable movement problems through partially analogous optimization strategies.

### 4.4 Correspondences in physical qualities and control capacity

Firstly, both Qinggong and parkour must be supported by strong lower-limb strength. The Qinggong “walking-in-a-basket” (5) training involves practicing in a large bamboo basket filled with sand and gravel to progressively enhance leg strength and endurance, ultimately facilitating lighter, more agile movement. Empirical studies indicate that parkour training can improve jumping performance (34); for example, practitioners show greater standing long-jump and vertical-jump scores compared with age-matched peers (35).

Secondly, parkour practitioners require strong core strength to maintain bodily stability during aerial vaulting and at the instant of landing, ensuring that movements are rapid and fluid (36). Qinggong practitioners, by contrast, emphasize with the trunk as the axis of rotation and integrating limb movements through trunk-centered force generation in order to maintain stable and agile movement form (37). These parallels suggest that both Qinggong and parkour require practitioners to possess strong proprioception and balance control, relying on the coordinated regulation of core musculature and lower limbs to control the position of the centre of mass during dynamic movements.

In addition, neural reaction speed is also a physical quality jointly emphasized in Qinggong and parkour. Qinggong has long been reputed for agile body technique; for example, when practising Qinggong on rugged terrain, practitioners must rapidly perceive footholds and adjust their footwork accordingly (30). This is analogous to parkour practitioners' immediate route judgement and avoidance of potential hazards within urban environments.

Finally, flexibility and impact control ability are also physical qualities emphasized by both Qinggong and Parkour. In modern training systems, good flexibility not only increases range of motion and movement expressiveness but may also reduce certain injury risks when flexibility is developed in relation to strength, control and task demands (38). Traditional Chinese martial arts have long emphasized the integration of hardness and softness; similarly, Qinggong training—which involves stretching of muscles and ligaments to enhance joint range of motion and tissue elasticity—regards flexibility training as a key component of the training regime (22).

### 4.5 Correspondences in training philosophies and methods

First, both practices make extensive use of the environment, forming an ecological training paradigm centered on real-world contexts. Unlike artistic gymnastics or track and field, which are conducted in standardized and homogenized training venues, parkour and Qinggong training take place in non-standardized real-world environments (1, 22). In terms of training methods, both eschew reliance on single, closed environments and instead pursue the enhancement of the organism's adaptability amid environmental variability.

Second, both Qinggong and parkour adhere to the principle of gradual progression. This is clearly reflected in Qinggong's training principle of “from static to dynamic, from easy to difficult, and from low to high”. The Qinggong secrets passed down by successive generations of Shaolin monks emphasize that practice should not seek quick results; one must follow a gradual progression, persevere consistently, and avoid sudden leaps in advancement (4, 5). This stepwise progression of difficulty is consistent with safety-oriented principles in contemporary parkour pedagogy, where foundational locomotor tasks, safe landing and rolling techniques, balance control, and low-height obstacle negotiation are typically developed before higher, faster, or more aerial movement tasks are introduced (34, 39, 40).

In terms of specific training methods, both practices make extensive use of repetitive practice. Traditional Chinese martial arts have long upheld the admonition to “practice a single move a thousand times” (41). The fundamental skills of Shaolin Qinggong require practitioners to perform repeated leg and waist strength exercises, thereby consolidating the foundation of physical qualities. Parkour studies and practitioner surveys indicate that parkour training also emphasizes repeated drilling of individual movements across varied contexts, which may reduce unnecessary movement variability and support the stabilization of adaptable movement patterns (1, 42, 43).

## 5 Implications of Qinggong training for parkour

This section moves from comparative interpretation to application, the following implications are presented as pedagogical hypotheses generated from the preceding analysis. Transfer from Qinggong sources to parkour coaching cannot be assumed automatically; it depends on progressive difficulty, qualified supervision, participant age and training background, site-specific risk assessment, and pilot testing of observable training variables. The aim is therefore to outline operationalisable cues and curriculum ideas that future studies or formal coaching programmes could evaluate.

### 5.1 Technical movements

#### 5.1.1 Adopt the “silent landing” buffering mechanism

The landings in Qinggong are called the “silencer of the walker” (5); their pursuit of “silent landing” is essentially a high-sensitivity proprioceptive feedback training, in which excessive noise may indicate inadequate energy dissipation and excessive impact force. Landing sound has been examined as a potential indicator of landing effectiveness, and experienced traceurs have shown lower maximal sound and lower kinetic loading variables than recreationally trained participants under some drop-height conditions (27). More generally, augmented auditory feedback can support motor learning when it supplies interpretable information about movement outcome or timing (44).

Accordingly, parkour coaching could explore “silent landing” as an auditory feedback cue for coordinating forefoot contact, lower-limb flexion sequencing, breath timing, and whole-body tension regulation, enables the exerciser to instinctively optimise the sequence of joint flexion, achieving a better cushioning effect. Potential operational indicators include landing sound amplitude, knee and hip flexion sequence, contact time, perceived stability, and whether the practitioner can continue into the next movement without an uncontrolled pause. This cue should be introduced first through low-height, low-speed tasks under coaching supervision before its use in faster or higher landings is empirically tested.

#### 5.1.2 Introduce the breakfall technique

For large vertical drops or landings with significant horizontal velocity, breakfall techniques in Qinggong serve as a potentially useful fall-management reference. These techniques exemplify Qinggong's alternation of soft and hard strategies (31), whereby impact that cannot be absorbed solely through joint flexion is redirected through tucked forward rolls, side rolls or backward-fall pathways. Rolls in Qinggong are not merely protective actions but also connecting movements, serving to establish a smooth transition between sudden stops and rapid motion (4, 5).

Parkour training could therefore further explore Qinggong-inspired side-fall, backward-fall, and oblique-roll patterns as supplementary drills. Potential training variables include roll direction, entry angle, shoulder/back contact path, exit speed, visual orientation following the roll, and movement continuity into the subsequent action. The transferability of these techniques is likely to depend on progressive exposure to increasing heights and movement velocities, initial practice on compliant surfaces before progression to harder surfaces, and appropriate qualified supervision. Their effects on safety and movement continuity remain hypotheses that require empirical verification in future studies.

#### 5.1.3 Draw on the “wall-stepping propulsion” techniques

In Chinese Martial Arts Qinggong (22), “Wall Stepping and Leaping” is mechanically deconstructed by emphasizing control of the “stepping tangential angle” (*α*). The Qinggong account suggests that wall-stepping is not merely an upward application of force, but an interaction between the wall's reaction force and friction generated by foot pressure. If the wall-stepping angle is too large (approaching a 90-degree vertical wall-step), the body will be pushed away from the wall surface by the rebound force; if the angle is too small, insufficient friction will lead to slipping. In parkour wall-run training, coaches often emphasize forceful stepping against the wall, but the precise interaction among approach angle, surface friction and foot-contact orientation remains a topic requiring empirical clarification (26, 45, 46).

Introducing a Qinggong-derived focus on the “stepping tangential angle”, together with assessment of wall-surface friction and approach speed, could be tested as a way to refine wall-run and rebound coaching. Observable variables would include approach angle, foot-contact height, foot orientation, slip/no-slip outcome, vertical displacement, and safe landing after wall contact. These drills should follow a gradual progression, moving from low walls and controlled surfaces to more complex environments.

### 5.2 Training methods

#### 5.2.1 Develop a combined buffering technique of “lifting Qi to lighten the body” and “fall-and-roll”

In parkour, high-drop descent actions occur frequently and impose large impacts on the lower-limb joints, thereby increasing injury risk when landing control is insufficient (47, 48). For this reason, Qinggong provides an internally controlled buffering concept, namely “lifting ‘Qi’ to lighten the body” and “sinking ‘Qi’ to the Dantian”, which involves coordinating the body's power generation through “Qi” (i.e., regulating the breath and attentional regulation). Relevant studies explain that during tasks requiring trunk extensor torque (such as jumping), breathing patterns interact with posture to influence abdominal muscle activation and intra-abdominal pressure; intra-abdominal pressure may contribute to trunk stabilization upon landing and provide support for the lumbar region (32, 33).

At the technical level, the Qinggong concept of “fall-and-roll” training (31) illustrates the martial arts approach to dissipating force through the principle of “softness overcoming hardness”. Such training could be adapted as a supplementary drill that broadens fall-management experience beyond the standard forward roll, including side falls, backward falls, and oblique rolling paths. The proposed value lies in distributing impact across a larger body surface and maintaining transition into subsequent movement. For operationalisation, the combined “breath-brace-roll” sequence could be broken into measurable components: preparatory exhalation or breath-hold strategy, trunk stiffness, landing sound, lower-limb flexion sequence, roll direction, contact surface and transition speed. Because the interpretation of “Qi” language is conceptual and abstract, these variables should be applied gradually and examined through pilot studies before stronger claims are made.

#### 5.2.2 Introduce balance training methods for multi-support environment simulation

Qinggong divides balance into static balance and dynamic balance (31). By repeatedly practicing on a variety of support surfaces, practitioners maintain bodily control in unstable or constrained environments and deeply develop the nervous system's agility and coordination, thereby improving practitioners' ability to perceive postural changes and the speed of muscular responses. However, the balance-support surfaces provided at parkour training sites are mostly standard railings or platforms, which are relatively fixed and predictable environments.

To this end, parkour training could cautiously introduce multi-support balance simulations, using various support surfaces such as cylinders, slopes, compliant platforms and irregular supports for walking, vaulting or low-height transition exercises. These variations stimulate adaptive postural adjustments by altering task and environmental constraints (39, 49), with the aim of enhancing perception of unstable support surfaces and improving postural adjustment during jumping and vaulting. Additionally, because human postural control depends on the dynamic weighting of visual, vestibular, and somatosensory information (50), visual perturbation training may be explored as one progression variable. For example, reduced visual preview, altered gaze demands or controlled distractions should be used only after stable performance on the basic task has been demonstrated, with the aim of encouraging coordinated visual-vestibular-proprioceptive adjustment rather than simply increasing difficulty.

Such varied-environment training should therefore be treated as a graded constraint-manipulation strategy. Potential operational indicators include support-surface width, height and compliance, visual preview time, error rate, balance-recovery steps, and successful completion of subsequent vaulting or landing tasks. It may enhance adaptability when facing uncertain obstacles, but its transfer to uncontrolled urban settings should be evaluated under context-specific safety protocols.

#### 5.2.3 Implement the “loading-unloading” periodized training model

Parkour training often emphasizes repeated bodyweight skill movements, environmental exploration, and self-directed or peer-supported practice; this is effective for technical familiarity, but may be insufficient if high-level performance development requires targeted strength, power, or load-management planning (42, 46). In contrast, Qinggong training possesses a rigorous resistance-training logic.

The *Jixiao Xinshu* (*New Treatise on Military Efficiency*) (30) records a progressive loading practice (e.g., wrapping sandbags around the legs and gradually increasing the weight, and then removing them while carrying out tasks to improve agility). From a contemporary perspective, this can be regarded as a form of post-activation potentiation enhancement (PAPE), whereby prior conditioning activities may acutely enhance subsequent explosive performance depending on load, rest interval, training status and task characteristics (51).

Accordingly, a Qinggong-inspired loading-unloading model could be proposed for empirical evaluation in parkour. During a foundational physical-preparation phase, carefully monitored weighted or resisted drills could be introduced within safe, standardized obstacle tasks; during high-skill sessions, loads would be removed so movement quality, timing, and landing control can be assessed. Potential operational variables include external load as a percentage of body mass, number of contacts or repetitions, rest interval between loading and unloaded movement, perceived exertion, jump or wall-run performance, and landing quality. For younger or novice participants, external loading should be introduced conservatively, if at all, and only after adequate bodyweight landing control has been established.

#### 5.2.4 Integrate the long-term training effects of “internal-external co-cultivation”

Qinggong emphasizes “internal-external co-cultivation” (3, 52), which can be considered a pedagogical logic in which internal regulation (breathing, attentional and affective control) and external technique (movement execution and spatial negotiation) are developed simultaneously through embodied practice.

Long-term, periodized external training can contribute to musculoskeletal adaptation through repeated mechanical loading. In general exercise science, mechanical loading can produce tendon adaptations, although the magnitude and mechanism depend on load strain, duration, tissue site, and population (53).

By contrast, the internal-regulation component does not refer to esoteric “internal power”, but to low-intensity somatic practices that coordinate breathing, posture, proprioceptive attention, and recovery. “Daoyin”, literally “guiding and stretching”, is commonly described as an ancient Chinese exercise form combining bodily movement, mental focus, and breathing (54). Slow-breathing and breath-focused movement practices may support autonomic regulation and attentional readiness, while posture–breathing coordination may contribute to abdominal muscle activation, intra-abdominal pressure management, and trunk organisation (33, 55, 56).

When integrated with proprioceptive, balance training and low-intensity active recovery, such internal-regulation work may help practitioners prepare arousal level, trunk control, and movement attention for subsequent obstacle-crossing tasks (57–59). Operationally, this internal-external pairing could be implemented as a low-intensity preparation or recovery component rather than as a claim of direct performance enhancement. Candidate measures include breathing frequency, perceived arousal, attentional focus, trunk-control quality and subsequent landing or balance performance.

### 5.3 Training system

#### 5.3.1 Integrated reconstruction of training philosophies and cultivation objectives

In its pedagogical transmission, traditional Chinese martial arts emphasize a complete and systematic structure. Practice is often conducted through a master-disciple model of oral instruction and embodied demonstration (60), in which the master teaches according to individual differences, progressing step by step from foundational skills and only transmitting advanced techniques once a solid base has been established. This cultivation experience, which integrates systematic organization, staged progression, and individualization, can support the continuity and safety of skill transmission.

Compared with the relatively formalized pedagogical hierarchy described in Qinggong sources, parkour instruction is diverse, moving across informal peer-led practice, classes, events, online resources, and formal coaching. Parkour studies describe collaborative learning and exploration as important features of training culture (42, 43, 46, 49). In formal coaching contexts, however, parkour instruction may vary in how it organizes progression, safety screening and individualized programming across regions and coaching settings. Therefore, drawing on the pedagogical experience of Qinggong to construct a systematic and staged framework for parkour training, supplemented by instructor guidance and individualized programming, may help strengthen consistency, safety awareness and scientific rigor of instruction without eliminating the exploratory character of parkour.

#### 5.3.2 Construct a three-dimensional training system of “foundation-specialization-practice”

In terms of curriculum design, Qinggong typically follows a pedagogical hierarchy of “foundation-specialization-practice” (3). First, the comprehensive abilities of students, such as strength, agility, and balance, are enhanced through solid foundational training. Subsequently, specialized Qinggong techniques are practiced in a targeted manner. Finally, various techniques are synthesized and applied in scenarios approaching real-combat situations to test and reinforce the learning. Existing research (29) has already scientifically categorized parkour technical movements into six major classes: flipping, hanging, balance, climbing, jumping, and synthesis, encompassing a total of 49 specific movements. This provides a basis for the modular layout of teaching content.

On this basis, the proposed three-level model should be understood as a staged curriculum structure rather than a direct transplantation of Qinggong pedagogy. The foundation stage corresponds to prerequisite movement literacy and risk-management skills: running mechanics, posture control, vault-landing, roll-buffering, lower-limb strength, trunk control, flexibility and basic balance. The specialization stage organizes typical parkour movements into modular clusters, including vaulting, hanging and climbing, jumping, rolling and balancing, so that coaches can match task difficulty to the learner's current action capabilities. The practice stage then places these modules into representative obstacle routes in which perception, decision-making and movement continuity are trained under progressively varied environmental constraints. This structure is compatible with ecological-dynamics accounts of parkour-style training environments and with recent pedagogical work treating parkour as a physical-education context for developing movement competence and creativity (39, 40, 46, 49, 61).

In formal educational or coaching contexts, a graded syllabus, movement checklist and safety protocol may help manage progression; this need not replace informal peer-led parkour learning cultures. Difficulty categories should therefore be used as optional pedagogical tools for sequencing practice and managing risk, not as universal standards for all parkour communities. Any rank-like system borrowed from martial arts would require local consultation, instructor training and empirical evaluation to ensure that it supports, rather than suppresses, the adaptive and exploratory logic of parkour (46, 49, 61, 62).

In practical terms, the framework suggests five cautious applications for formal parkour coaching: using landing sound as a feedback cue, diversifying fall-management drills, manipulating support-surface and visual constraints, planning load-unload sequences only after bodyweight competence, and organizing teaching through prerequisite, specialized and integrative tasks. These applications should be introduced progressively, recorded through observable indicators and evaluated through pilot studies before being generalized across ages, training levels or cultural settings.

## 6 Discussion and limitations

### 6.1 Discussion

This paper reviews traditional Qinggong and parkour through a functional and pedagogical comparative lens. The analysis suggests that, despite evident cultural differences, the two practices address comparable obstacle-crossing problems and display useful correspondences in movement organization, landing-buffering ideals, balance adaptation, progressive training, and staged skill development. On this basis, Qinggong's training ideas may offer a reference point for rethinking selected aspects of parkour pedagogy.

Practically, the model is best read as a design proposal for formal educational or coaching settings rather than as a fixed curriculum for all parkour communities. Coaches may begin with low-height landing cues and fall-management tasks, add controlled balance and environmental variability, and finally integrate movement modules into representative routes. Each step should be matched to the learner's background and assessed through observable outcomes before progression.

Theoretically, the comparison contributes to cross-cultural movement studies by showing how traditional body techniques can be mobilized as heuristic resources for contemporary pedagogy. What travels across cultures in the present argument is a set of interpretable movement problems, training ideals, and pedagogical hypotheses that can be reconfigured in contemporary coaching. The value of the comparison therefore lies in heuristic translation across movement cultures: Qinggong textual and practical traditions may help generate questions for parkour training design.

### 6.2 Limitations and future research

Several limitations should be acknowledged. First, this study is a conceptual comparative analysis based on purposive and theoretical sampling, not a systematic review or experimental intervention. The selected sources were chosen for conceptual relevance to obstacle-crossing, landing, balance, parkour pedagogy, and Qinggong training, which means that other relevant materials may not have been included.

Second, the Chinese materials used in the analysis differ in genre, period, and evidentiary status. Martial arts manuals, historical writings, later reconstructions, performance representations, and literary-cultural images cannot be treated as equivalent forms of evidence. They can illuminate technical ideals, pedagogical norms, and symbolic meanings, but they cannot by themselves establish technical efficacy or historical influence.

Third, the proposed correspondences remain interpretive. The paper identifies comparable movement problems and proposes training hypotheses, but it does not demonstrate that Qinggong-derived drills improve parkour performance, reduce injury incidence, or transfer reliably across learners.

Fourth, translation itself is a limitation. Terms such as “Qing”, “Gong”, “Qi”, and “internal-external co-cultivation” carry cultural, medical, martial, and pedagogical meanings that cannot be fully captured by single English equivalents. The article therefore uses functional explanation rather than direct equivalence, but some semantic complexity is inevitably reduced in the process.

Future studies should proceed in several directions. Historiographical research could examine Qinggong manuals, martial arts lineages, performance traditions, and modern representations with more detailed source criticism. Biomechanical research could test selected Qinggong-inspired drills, such as silent-landing feedback, fall-and-roll variations, constrained-support balance tasks, or wall-stepping propulsion, using motion capture, force platforms, electromyography, and injury-surveillance designs. Pedagogical research could then evaluate whether the foundation-specialization-practice framework improves learning progression, safety awareness, movement creativity, and environmental adaptability among children, adolescents, and adult practitioners in different coaching contexts.
